# Death from Chloroform

**Published:** 1861-01

**Authors:** 


Death from Chlorgform.—Again we have to record a case of death from chloroform occuring in Bellvue Hospital, in New York, during a surgical operation, and offering another forcible plea for the abandonment of chloroform and the substitution of *ether* in surgical practice, except in cases where ether has been tried and found to be incapable of producing anæsthesia.

In this case, as indeed in many others, the chloroform was administered, so the verdict says, with the utmost caution, yet suddenly the patient gave a few convulsive respirations and immediately expired. All efforts to restore him were unavailing.

Why should chloroform be continued to be given when its dangers have been so amply and so fearfully demonstrated and when in ether we have an article nearly as certian, although acting a little slower, but known to be attended with far less risk of life? It is true, we know, that it is somewhat more tedious process to produce anæsthesia by ether than by chloroform ; yet is there any surgeon's time so valuable that at the risk of his patient's life it must be used in preference to a safer article?

These are the questions which force themselves upon our attention, when again and again we are obliged to refer in our columns to "*deaths from chloroform.*" Let the profession ponder them well —*Med. and Sur. Rep.*

